# The complete mitochondrial genome of the greater green snake *Cyclophiops major* (Reptilia, Serpentes, Colubridae)

**DOI:** 10.1080/23802359.2017.1331326

**Published:** 2017-05-27

**Authors:** Hongji Sun, En Li, Long Sun, Peng Yan, Hui Xue, Fang Zhang, Xiaobing Wu

**Affiliations:** Key Laboratory for Conservation and Exploitation of Biological Resources of Anhui Province, College of Life Sciences, Anhui Normal University, Wuhu, P.R. China

**Keywords:** Mitochondrial genome, *Cyclophiops major*, Colubridae

## Abstract

The greater green snake *Cyclophiops major* is a protected and colubrid species. Here, we investigated the complete mitochondrial genome of *C. major*. The genome is 17,217bp in size, including 13 protein-coding genes, 2 rRNA genes, 22 tRNA genes, 2 control regions, and an origin of light-strand replication. All genes are distributed on the heavy strand, except for ND6 gene and 8 tRNA genes. The AT content of the overall base composition of light strand is 59.83%, showing AT bias. Phylogenetic tree was built based on the genome of *C. major* and other related snakes to analyze their phylogenic relationship.

The greater green snake *Cyclophiops major* is a protected and docile species, and is considered non-venomous, belonging to the genus *Cyclophiops* of family Colubridae, which is widely distributed in China, Vietnam, and eastern Lao PDR (Orlov et al. 2000; Dieckmann et al. 2014; Ziegler et al. 2014). In this study, we determined the complete mitochondrial genome (mitogenome) of *C. major* (The GenBank accession number: KF148620). Samples of *C. major* (RE03064) were collected from Huang mount, Anhui Province of China, and deposited in the laboratory of the College of Life Sciences of Anhui Normal University, Wuhu, China. The total length of *C. major* is 17,217 bp. It consists of 13 protein-coding genes (PCGs), 2 ribosomal RNA (rRNA) genes, 22 transfer RNA (tRNA) genes, 2 control regions (CRs, CRI and CRII), and an origin of light-strand replication (O_L_), which is similar to other reported alethinophidian snakes (He et al. 2010; Jang & Hwang, 2011; Li et al. 2016; Qian et al. 2016). The overall base composition of the light strand is as follows: A (34.61%), T (25.22%), C (27.89%), and G (12.28%), the AT content (59.83%) is significantly higher than the GC content (40.17%), which shows AT bias.

Except for ND6 gene and 8 tRNA genes, other genes are encoded by the heavy strand. Most PCGs use ATG as the start codon, while ND1, ND2, ND3 and COI genes use ATA, ATC, ATT and GTG as the start codon, respectively. Seven PCGs were terminated with the complete stop codon, ATP6, ATP8, ND4 and ND4L ended with TAA, COI gene stops with AGG, ND5 and ND6 using TAG as the stop codon, whereas other 6 PCGs ended with incomplete stop codon with T.

The length of the 12S rRNA gene is 923 bp, and is located between tRNA^Phe^ and tRNA^Val^ gene. 16S rRNA gene is 1478 bp, and is located between tRNA^Val^ and ND1. There are 22 tRNA genes in *C. major* mitogenome, with the size ranging from 57 bp in tRNA^Ser(AGY)^ to 73bp in tRNA^Leu(UUR)^. The sequence length of the CRI and CRII is 1050 and 1049 bp, respectively. The CRI is surrounded by tRNA^Pr^° and tRNA^Phe^, while the CRII is located between tRNA ^Ile^ and tRNA^Leu(UUR)^. The O_L_ is located between tRNA^Asn^ and tRNA^Cys^ gene, with a length of 36 bp in the WANCY cluster.

We used Mega6 (http://megasoftware.net) to build the neighbor-joining phylogenetic tree (Figure 1), including mitogenome of *C. major* and other closely related 12 species that are from the family Colubridae, Acrochordidae, Elapidae, and Viperidae, which belong to Serpentes. *Alligator sinensis* was set as an outgroup. The results showed that *C. major* was clustered with the *Z. dhumnades*, highly supported by a bootstrap value of 100, and then clustered with *O. taeniurus, O. rufodorsatus, E. Poryphyracea, H. Chlorophaea, S. Collaris,* which supported the data that mitogenome of *C. major* are more closely related to other species of Colubridae family. The present study will provide a foundation for further research on the population genetics and systematic analyses of *C. major*, and provide genomic resources for Colubridae studies.

**Figure 1.**
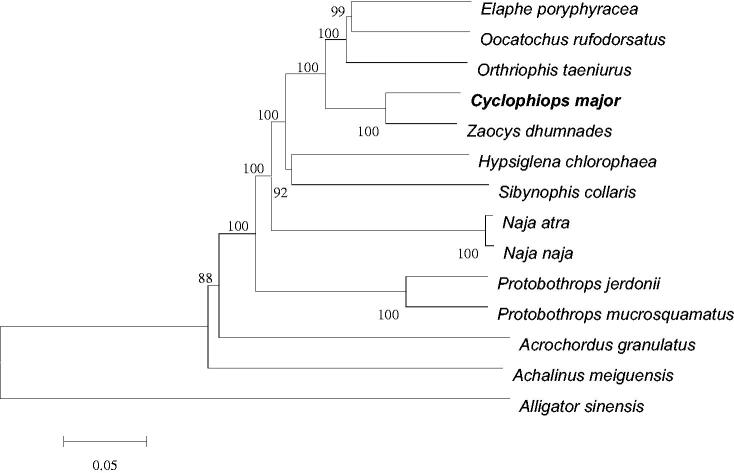
Neighbor-joining phylogenetic tree of complete mitogenome of *C. Major* and other related 12 species was constructed, with *Alligator sinensis* as an outgroup. The numbers on the each node branches are bootstrap values. All species’ accession numbers are listed as follows: *Cyclophiops major* KF148620; *Zaocys dhumnades* KF148621; *Oocatochus rufodorsatus* KC990020; *Orthriophis taeniurus* KC990021; *Achalinus meiguensis* NC_011576; *Elaphe poryphyracea* NC_012770; *Hypsiglena chlorophaea* NC_013977; *Sibynophis collaris* NC_016424; *Acrochordus granulatus* NC_007400; *Naja atra* NC_011389; *Naja naja* NC_010225; *Protobothrops jerdonii* NC_021402; *Protobothrops mucrosquamatus* NC_021412; *Alligator sinensis* NC_004448.

## References

[CIT0001] Dieckmann S, Norval G, Mao JJ. 2014 Notes on the reproductive biology of the greater green snake, Cyclophiops major (Günther, 1858), in Taiwan. IRCF Reptiles Amphib. 21:100–102.

[CIT0002] He M, Feng JC, Zhao EM. 2010 The complete mitochondrial genome of the Sichuan hot-spring kell-back (*Thermophis zhaoermii*; Serpentes: Colubridae) and amitogenomic phylogeny of the snakes. Mitochondrial DNA. 21:8–18. 20085537 10.3109/19401730903505867

[CIT0003] Jang KH, Hwang UW. 2011 Complete mitochondrial genome of the black-headed snake *Sibynophis collaris* (Squamata, Serpentes, Colubridae). Mitochondrial DNA. 22:77–79. 22040070 10.3109/19401736.2011.624601

[CIT0004] Li E, Sun FX, Zhang RD, Chen J, Wu XB. 2016 The complete mitochondrial genome of the striped-tailed rat-snake, *Orthriophis taeniurus* (Reptilia, Serpentes, Colubridae). Mitochondrial DNA. 27:599–600. 24730609 10.3109/19401736.2014.908364

[CIT0005] Orlov NL, Murphy RW, Papenfuss TJ. 2000 List of snakes of Tam-Dao mountain ridge (Tonkin, Vietnam). Russ J Herpetol. 7:69–80.

[CIT0006] Qian LF, Zhang CL, Huang X, Pan T, Wang H, Zhang BW. 2016 Mitochondrial genome of *Dinodon rufozonatum* (Squamata: Colubridae: Dinodon). Mitochondrial DNA. 27:970–971. 24937574 10.3109/19401736.2014.926510

[CIT0007] Ziegler T, Tran DTA, Nguyen TQ, Perl RGB, Wirk L, Kulisch M, Lehmann T, Rauhaus A, Nguyen TT, Le QK, Vu TN. 2014 New amphibian and reptile records from Ha Giang Province, northern Vietnam. Herpetol Notes. 7:185–201.

